# Differently Pre-treated Rapeseed Meals Affect *in vitro* Swine Gut Microbiota Composition

**DOI:** 10.3389/fmicb.2020.570985

**Published:** 2020-08-28

**Authors:** Cheng Long, Sonja de Vries, Koen Venema

**Affiliations:** ^1^Faculty of Science and Engineering, Centre for Healthy Eating and Food Innovation, Maastricht University, Maastricht, Netherlands; ^2^School of Nutrition and Translational Research in Metabolism (NUTRIM), Maastricht University, Maastricht, Netherlands; ^3^Animal Nutrition Group, Wageningen University & Research, Wageningen, Netherlands

**Keywords:** rapeseed meal, pig gut microbiota, cellulase, pectinase, alkaline, adaptation period

## Abstract

The aim of the study was to investigate the effect of untreated and processed rapeseed meal (RSM) on fiber degradability by pig gut microbiota and the adaptation of the microbiota to the substrate, by using the Swine Large Intestine *in vitro* Model (SLIM). A standardized swine gut microbiota was fed for 48 h with pre-digested RSM which was processed enzymatically by a cellulase (CELL), two pectinases (PECT), or chemically by an alkaline (ALK) treatment. Amplicons of the V3–V4 region of the 16S rRNA gene were sequenced to evaluate the gut microbiota composition, whereas short chain fatty acids (SCFA) were measured to assess fiber degradation. Adaptive gPCA showed that CELL and ALK had larger effects on the microbiota composition than PECT1 and PECT2, and all substrates had larger effects than CON. The relative abundance of family Prevotellaceae was significantly higher in CELL treatment compared to other treatments. Regardless of the treatments (including CON), the relative abundance of *Dorea*, *Allisonella*, and *FamilyXIIIUCG_001* (in the order of Clostridiales) were significantly increased after 24 h, and *Parabacteroides*, *Mogibacterium*, *Intestinimonas*, *Oscillibacter*, *RuminococcaceaeUCG_*009, *Acidaminococcus*, *Sutterella*, and *Citrobacter* were significantly higher in abundance at time point 48 compared to the earlier time points. *Prevotella* 9 had significant positive correlations with propionic and valeric acid, and *Mogibacterium* positively correlated with acetic and caproic acid. There was no significant difference in SCFA production between untreated and processed RSM. Overall, degradability in the processed RSM was not improved compared to CON. However, the significantly different microbes detected among treatments, and the bacteria considerably correlating with SCFA production might be important findings to determine strategies to shorten the fiber adaptation period of the microbiota, in order to increase feed efficiency in the animal, and particularly in pig production.

## Introduction

Rapeseed meal (RSM) is not only an important alternative feed ingredient for protein-rich feeds (where mainly soybean meal is used as protein source), but RSM is also rich in non-starch polysaccharides (NSP) (Pustjens, 2013; De Vries et al., 2014; Pustjens et al., 2014a). However, a disadvantage of RSM is that a high proportion of cell wall polysaccharides cannot be utilized by endogenous enzymes from monogastric animals, and the NSP can affect the digestion of other nutrients by physical hindrance or physiological shift in the intestine (e.g., increasing digesta viscosity) (De Vries et al., 2014). NSP can only be fermented in the swine large intestine by the gut microbiota, and research has shown that the pig gut microbiota needs an adaptation period to express its maximum enzymatic potential after a dietary change (Castillo et al., 2007). However, most of the scientific research focused on how the fibers status affected nutrient digestibility or animal performance during the adaptation period (Huang et al., 2018; Lyu et al., 2018), and few monitored how the gut microbiota changed during this period (Umu et al., 2018).

Previous studies showed that a high-fiber rapeseed diet did not results in a significant increase in SCFA content in the chyme of RSM-fed pigs after a 3-week adaptation period (Chen et al., 2018; Umu et al., 2018). However, SCFA-producing microbes, such as *Dialister*, *Shuttleworthia*, *Bulleidia*, *Coprococcus*, and *Lachnospira*, were detected more abundant in the colon of high-fiber rapeseed pigs compared to those in the control pigs (Umu et al., 2018). This might be because the 3-week adaptation period was too short to have a considerable change in the metabolic function of SCFA-producing microbes, or more likely, the SCFA were rapidly utilized by the intestinal epithelium of the host. Adaptation duration in pigs supplemented with 19.5% palm kernel showed that 3 weeks of adaptation is needed, and a longer adaptation time is suggested as dietary palm kernel content of the diet increases (Huang et al., 2018). Huang et al. (2018) also observed that apparent total tract digestibility of acid detergent fiber significantly increased when the adaptation time increased from 7 to 28 days (Huang et al., 2018). Another report showed that microbial cellulase activity was only observed after a 6-week adaptation period when pigs were fed with different types of dietary fiber (8% sugar beet pulp, or 10% wheat bran) (Castillo et al., 2007). Montoya et al. (2018) demonstrated that an optimum adaptation time also depends on the dietary level when growing pigs were fed with kiwi fruit (Montoya et al., 2018). Thus, the optimal adaptation time should be evaluated case by case.

Treatment with carbohydrases, such as cellulase and pectinase, have been reported to improve the NSP degradation after a 4-week adaptation in both boilers and *in vitro* (Pustjens et al., 2012; De Vries et al., 2014). Thus, in the current study, RSM (predigested) was treated independently with two kinds of pectinases (PECT1 and PECT2), one cellulase (CELL), or alkaline (ALK), and afterward the untreated and treated RSM preparations were fed to the recently developed Swine Large Intestine *in vitro* Model (SLIM) (Long et al., 2020) for a 48 h period. SLIM was established on the basis of the human, computer-controlled, dynamic TNO *in vitro* model of the colon, TIM-2 (Minekus et al., 1999), which accurately simulates the physiological conditions in the lumen of the human proximal colon, containing a gut microbiota of human origin. A unique design of the machine is that the model is equipped with a dialyzate system, which prevents accumulation of microbial metabolites which would lead to the inhibition or death of microbes in the model when they would accumulate. Since all metabolites (i.e., SCFA) are collected, and basically a mass-balance can be made, this allows for real determination of the production of microbial metabolites such as SCFA, which is not possible *in vivo*.

In the current study, the effects of processed rapeseed meal on swine gut microbiota and SCFA production were investigated. We hypothesized that (1) the treatments on RSM will increase SCFA production (fiber degradability) during the fiber fermentation period compared to when the swine gut microbiota is fed with untreated RSM, and (2) the effects of processed RSM on the swine gut microbiota are different according to the treatments. The results of the current study give insight into how processed RSM affect pig gut microbiota during the fermentation period, which is important information to use to shorten the feed adaptation period and to improve feed efficiency.

## Materials and Methods

### Substrate Preparation

**FIGURE 1:**
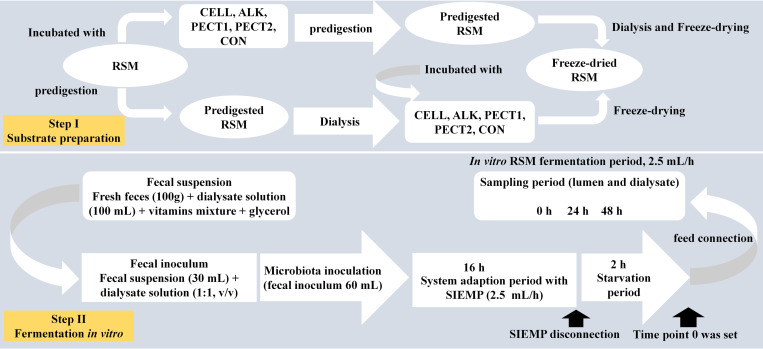
Schematic presentation of experimental setup for fiber fermentation in the Swine Large Intestine *in vitro* model (SLIM).

Rapeseed meal (Brassica napus, Cargill N.V., Antwerp, Belgium; 2011) was obtained from a commercial feed mill (Agrifirm B.V., Utrecht, Netherlands). Preparation method I (predigesting RSM after carbohydrase or alkaline treatment; Figure 1): to 200 g of substrate 40 mL 10^∗^gastric electrolyte concentrate solution (GES, 310 g sodium chloride, 110 g potassium chloride, 15 g calcium chloride di-hydrate, and 4840 g ultrapure water) and 360 mL ultrapure water were added. The pH was adjusted to 5.5 and then nothing (CON), ALK (6 M NaOH) or 10 mL of the following carbohydrases were added; CELL, Accellerase 1000 (Sigma-Aldrich, Missouri, United States); PECT1, Pectinex Ultra SP (Novozymes A/S, Bagsvaerd, Denmark); or PECT2, Multifect Pectinase (DuPont Industrial Biosciences, Genencor division, Rochester, NY). Enzyme preparations were incubated at 37°C for 2 h, with occasional shaking (every 30 min), while ALK was incubated overnight at 4°C. Enzyme preparations were then heated at 100°C for 5 min to inactive enzymes. Afterward, for all five samples, 120 mL GES was added and the pH adjusted to 3 to continue with the gastric incubation according to the pre-digestion protocol as described elsewhere (Sáyago-Ayerdi et al., 2017). After pre-digestion, the slurry was centrifuged (8.000 g, at 4°C, for 20 min) and dialysis was performed for the supernatants. For dialysis, a dialysis membrane (Sureflux, Nipro Europe Group Companies, Mechelen, Belgium) was used with a peristaltic pump to remove small digestion products and water. After reduction of the total volume to ∼450–500 mL, supernatant was mixed with pellet, and freeze-dried. Method II (predigesting RSM before carbohydrase or alkaline treatment; Figure 1): four batches of 200 g RSM were predigested as described before (Sáyago-Ayerdi et al., 2017) and then dialyzed. Afterward, 55 mL 10^∗^GES was added, and pH adjusted to 5.5, after which 10 mL of CELL, PECT1, PECT2, or ALK treatment commenced, respectively. Enzyme preparations were incubated at 37°C for 2 h with occasional shaking (every 30 min), and ALK was incubated overnight at 4°C. Afterward, enzyme preparations were heated at 100°C for 5 min to inactive enzymes, and pH was neutralized to 6.5–7 with HCl or NaOH, and the samples were freeze-dried.

### Fermentation in the Swine *in vitro* Large Intestinal Model (SLIM)

The setup of SLIM was as follows: a fully computer-controlled *in vitro* model based on TIM-2 (Minekus et al., 1999) was used to mimic the swine large intestine (Supplementary Figure S1). The pH (5.9) was controlled by continuous addition of 2 M sodium hydroxide. Standard ileal efflux medium of pigs (SIEMP) was used to simulate the materials entering the colon. The SIEMP, adapted from Gibson et al. (1988) and described in Long et al. (2020), contained the following components (g/L): 74.6 maize starch, 9.0 xylan, 19.0 pectin, 9.0 amylopectin, 9.0 arabinogalactan, 9.0 arabinoxylan, 9.0 xyloglucan, 31.5 Tween 80, 43.7 casein, 0.7 ox-bile, 43.7 bactopepton, 4.7 K_2_HPO_4_.3H_2_O, 0.009 FeSO_4_.7H_2_O, 8.4 NaCl, 0.8 CaCl_2_.2H_2_O, 0.7 MgSO_4_.7H_2_O, 0.05 bile, 0.02 hemin, and 0.3 cysteine⋅HCl, plus 1.5 mL of a vitamin mixture containing (per liter): 1 mg menadione, 0.5 mg vitamin B12, 2 mg D-biotin, 10 mg pantothenate, 5 mg p-aminobenzoic, 4 mg thiamine and 5 mg nicotinamide acid. The pH was adjusted to 5.9. SIEMP only contains indigestible carbohydrates and hence did not require pre-digestion. Dialysis liquid contained (per liter): 2.5 g K_2_HPO_4_.3H_2_O, 0.005 g FeSO_4_.7H_2_O, 4.5 g NaCl, 0.45 g CaCl_2_.2H_2_O, 0.05 g bile, 0.5 g MgSO_4_.7H_2_O and 0.4 g cysteine⋅HCl, plus 1 mL of the vitamin mixture. All medium components were purchased at Tritium Microbiology (Eindhoven, Netherlands). The pig fecal inoculum was a standardized microbiota from growing pigs (48 pens with 6 pigs/pen, Hypor Libra x Hypor Maxter, Hendrix Genetics, Boxmeer, Netherlands), consisting of pooled freshly collected feces from the floor, but only material from the top (so not toughing the floor) was selected. Since this was not an intervention study, feces collection from the floor of the pins of the pigs did not require ethical approval in accordance with local/national guidelines.

In order to create a complete anaerobic environment, SLIM with 90 mL dialyzate in each unit was flushed with gaseous nitrogen for at least 3 h before incorporating the standardized microbiota (Supplementary Figure S1). Thirty mL of the standardized microbiota was added to the system, making the total volume of each SLIM-unit 120 mL. The microbiota was adapted to the model with SIEMP for 16 h. During the adaptation phase (Figure 1) SIEMP was administered at 60 mL d^–1^ [corresponding to 7.5 g carbohydrate d^–1^] (de Souza et al., 2014) through the feeding syringe (Supplementary Figure S1). At the end of the adaptation period, a 2-h starvation period was performed (Figure 1), which was used to allow all the carbohydrates within SIEMP to be fermented by the microbiota and after this time point 0 was set. Afterward, the ‘fiber adaptation’ period (48 h) was performed, in which the microbiota was allowed to adapt to the test products (CON, CELL, PECT1, PECT2 and ALK). During this stage, carbohydrates in SIEMP were replaced with 7.5 grams of (treated) RSM which were added continuously in the model at a rate of 2.5 mL/h for 48 h (Figure 1), corresponding to 7.5 g carbohydrate d^–1^.

### Sample Collection

Samples (*n* = 2) from lumen and spent dialyzate were collected at time point 0, 24, and 48 h (t0, t24, and t48). They were snap-frozen in liquid nitrogen and stored until analyses. Lumen samples were used to analyze microbiota composition, and both lumen and dialysis samples were analyzed for short chain fatty acid (SCFA) concentrations.

### Sequencing of V3–V4 Region of the 16S rRNA Gene

Microbial DNA extraction and sequencing of the V3-V4 region of the 16S rRNA gene were performed by BaseClear B.V. (Leiden, Netherlands). Briefly, genomic DNA extraction was performed using the Quick-DNA^TM^ Fecal/Soil Microbe Miniprep Kit (Zymo Research, CA, United States) according to the manufacturer’s instructions. Barcoded amplicons from the V3-V4 region of 16S rRNA genes were generated using a 2-step PCR. 10–25 ng genomic DNA was used as template for the first PCR with a total volume of 50 μL using the 341F (5′-CCTACGGGNGGCWGCAG-3′) and the 785R (5′-GACTACHVGGGTATCTAATCC-3′) primers (Klindworth et al., 2013) appended with Illumina adaptor sequences. PCR products were purified (QIAquick PCR Purification Kit, Venlo, Netherlands) and the size of the PCR products were checked on a Fragment analyzer (Advanced Analytical, Ankeny, United States) and quantified by fluorometric analysis. Purified PCR products were used for the 2nd PCR in combination with sample-specific barcoded primers (Nextera XT index kit, Illumina, CA, United States). Subsequently, PCR products were purified, checked on a Fragment analyzer and quantified, followed by multiplexing, clustering, and sequencing on an Illumina MiSeq with the paired-end (2x) 300 bp protocol and indexing. The sequencing run was analyzed with the Illumina CASAVA pipeline (v1.8.3) and demultiplexed based on sample-specific barcodes. Raw sequencing data was submitted to the European Nucleotide Archive under the accession number: PRJEB38485.

### Bioinformatics Analysis

The demultiplexed raw sequences obtained from BaseClear were processed using QIIME2 pipeline (Bolyen et al., 2019). In short, reads were imported, quality filtered and dereplicated with q2-dada2 (Callahan et al., 2016), after which dada2 was performed with paired-end reads and truncations parameters as follows:the first 10 base pairs were trimmed off and at position 280 base pairs the fragment was truncated in forward reads, and at position 240 base pairs for the reverse reads. The processed sequences were used for all the downstream analyses. Alpha-diversity (Shannon index) and beta diversity [weighted and unweighted UniFrac; (Lozupone and Knight, 2005; Lozupone et al., 2007)] were analyzed by the q2-phylogeny plugin (https://github.com/qiime2/q2-diversity).

#### Adaptive Generalized PCA, gPCA

Adaptive gPCA (Fukuyama, 2017; Fukuyama et al., 2017) was performed on the ASV-tables generated in QIIME. This is a relatively new method which can obtain a low-dimensional representation of the samples in which the axes are interpretable at a fine phylogenetic scale, which can be used to compare the dissimilarities between samples more accurately than that of UniFrac. The mathematical algorithm for gPCA can be found in the literature (Fukuyama, 2017). R packages biomformat, yaml, Biostrings, phyloseq, Hmisc, qiiime2R, vegan, ggplot2, and adaptiveGPCA were used in this analysis.

#### Phylogenetic Investigation of Communities by Reconstruction of Unobserved States, PICRUSt2

PICRUSt2 software (Douglas et al., 2019) was used to predict microbial functional abundances based on marker gene sequences. KEGG database was used to predict the results.

### Chemical Analyses

#### Short-Chain Fatty Acids Analyses

Samples from lumen and dialyzate were analyzed by Brightlabs (Venlo, Netherlands) for determination of concentrations of SCFA (including the middle chain fatty acids valeric and caproic acid). Ion exclusion chromatography (IEC) was applied on an 883 Ion Chromatograph (IC; Metrohm, Herisau, Switzerland), using a Transgenomic IC Sep ICE-ION-300 column (30 cm length, 7.8 mm diameter and 7 μm particles) and a MetroSep RP2 Guard. The mobile phase consists of 1.5 mM aqueous sulfuric acid. A column flow rate of 0.4 ml min^–1^ was used. The temperature of the column was 65°C. The organic acids were detected using suppressed conductivity detection. Samples were centrifuged (14,000 rpm, 10 min), and the clear supernatant was filtered through a 0.45 μm PFTE filter and diluted with mobile phase (for lumen 1:5, for dialyzate 1:2). Ten microliters were loaded on the column by an autosampler 730 (Metrohm). Molecules were eluted according to their pKa.

### Statistical Methods

Kruskal-Wallis Rank Sum Test was applied to compare alpha diversities (Shannon index) among different RSM treatments and time points, and Wilcoxon Rank Sum Test was used for pairwise comparison in R version 3.5.3 (https://www.r-project.org/). Bonferroni was used to correct *P*-values. Permutational multivariate analysis of variance [PERMANOVA; (Anderson, 2005)] was performed to test the significance of beta diversity (weighted and unweighted UniFrac) between non-processed and processed RSM in QIIME2. The results were visualized in R. Linear discriminant analysis effect size [LEfSe] (Segata et al., 2011) was used to find biomarkers between groups using relative abundances from the feature tables generated in QIIME at genus level.

Pearson correlations between continuous meta-variables (SCFA production) and taxonomic variables were calculated and visualized in R. Parameters were set as follows: Missing values for meta-variables were handled as NO imputation (replacing missing data with substituted); zeros were kept for the calculation of correlation; a minimum relative abundance of 0.1% was considered for calculation; a minimum of 4 pairs of observations were required for calculation of correlations.

Kruskal-Wallis Rank Sum Test was conducted to compare SCFA production among CON, ALK, PECT1, PECT2, and CELL in the built-in R package.

## Results

### RSM With Different Treatment Methods Differentially Changed the Microbial Phylogenetical Relationship During the Fiber Fermentation Period

There were 26839 ± 4458 (ALK), 26973 ± 5187 (CELL), 26243 ± 4871 (PECT1), 29597 ± 4026 (PECT2), and 52927 ± 4055 (CON) raw sequences obtained after Illumina Miseq sequencing. Filtered reads were 22612 ± 4162, 22862 ± 4408 22612 ± 4162, 23895 ± 2308, and 40092 ± 4147 sequences in ALK, CELL, PECT1, PECT2, and CON, respectively. And finally 14106 ± 2470, 14665 ± 3831, 13265 ± 2686, 15332 ± 1814, and 21551 ± 2348 non-chimeric sequences were obtained for ALK, CELL, PECT1, PECT2, and CON, respectively, after quality control. Finally, 1261 ASVs were detected.

**FIGURE 2:**
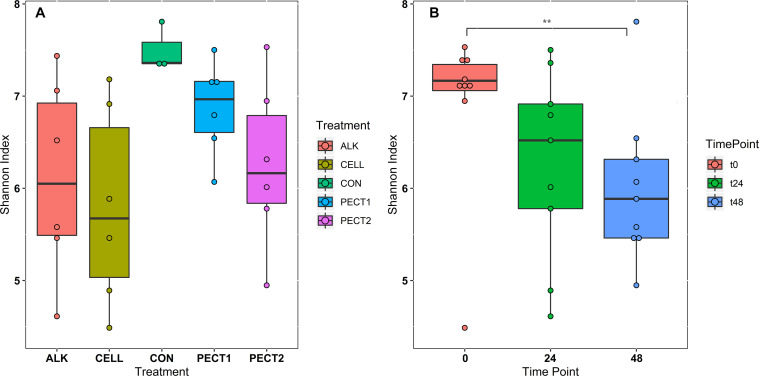
Community α-diversity represented by Shannon index from each treatment **(A)** and time point **(B)**. The treatments are non-processed RSM (CON) and RSM processed by Accellerase 1000 (CELL), Pectinex Ultra SP (PECT1), Multifect Pectinase (PECT2), or 6 M NaOH (ALK).

**FIGURE 3:**
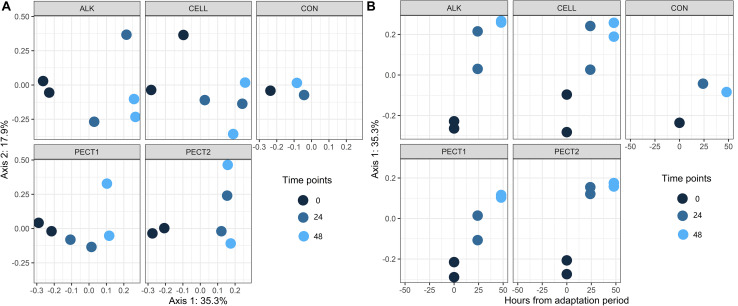
Two views of results from adaptive gPCA. **(A)** shows the sample scores from gPCA plotted on the first two axes. **(B)** sample scores have been centered by treatments so as to better show the within-treatment variation, and the centered scores along the first axis are displayed over time.

There were no significant difference in α-diversity (Shannon Index, Ace, Chao1 and Simpson) between non-processed RSM (CON) and processed RSM (ALK, PECT1, PECT2, and CELL) (Figure 2A and Supplementary S2). Shannon Index, Ace and Chao1 were significantly decreased during the fiber fermentation period (Figure 2B and Supplementary S2), significant at 72 h. Unweighted UniFrac shows that CON had a significant different (*P* = 0.02) microbial community compared to ALK, PECT1, PECT2, and CELL while there were no significant difference (*P* = 0.55) between non-processed and processed RSM in terms of weighted UniFrac (Supplementary Tables S1, S2). The biplot of unweighted UniFrac (Supplementary Figure S3) showed that genus *Prevotella* 9 was mainly associated with t0, while *Ruminococcaceae gauvreauii* group, *Eubacterium nodatum* group, *Succiniclasticum*, *Mogibacterium*, *Olsenella*, *Prevotellaceae NK3B31* group *Bacteroidles S24-7* group *Rikenellaceae* RC9 gut group, and *Ruminococcaceae UCG-002* were associated with t24 and t48. Afterward, adaptive gPCA (Fukuyama et al., 2017) was used to identify the dissimilarities among samples. The results show that the starting points (t0) of all the treatments were approximately located in the same spot of the principle plane, and the later time points lay along the first adaptive gPCA axis (Figure 3A). After 48 h adaptation, the microbiota composition changed considerably in the processed RSM groups compared to the start time points, whereas CON only showed a slight difference between t48 and t0 (Figure 3B).

Alpha-diversity (Shannon index) and β-diversity (weighted and unweighted UniFrac) showed that there were no significant differences between microbiota fed with modified RSM before or after dialysis (data not shown). These indicated that predigesting before or after modifying RSM by ALK or enzymes did not differentially affect microbiome composition. Our previous study showed that predigesting before or after processing RSM had little effect on their monosaccharide constitution (Long et al. manuscript2 submitted).

### Fermentation Time Had Larger Effect on the Microbiota Composition During the Fiber Fermentation Period Than the Treatments on RSM Did

**FIGURE 4:**
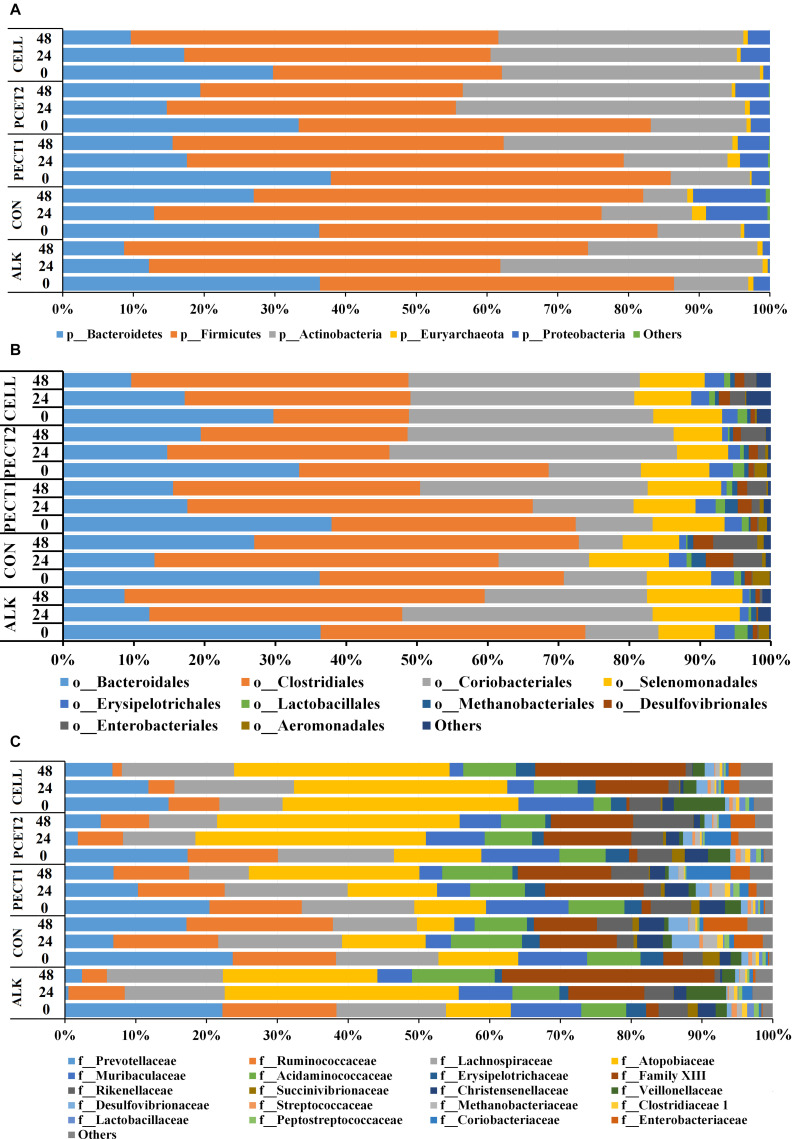
Relative abundances of microbial phyla **(A)**, orders **(B)**, and families **(C)** in pig microbiomes fed with non-processed RSM (CON) or RSM processed by Accellerase 1000 (CELL), Pectinex Ultra SP (PECT1), Multifect Pectinase (PECT2), or 6 M NaOH (ALK) at different time points (0, 24, and 48 h).

Figure 4A and Supplementary Figure S4A show at the phylum-level that the relative abundances of Bacteroidetes in response to ALK, CON, PECT1, PECT2, and CELL were quite similar to each other at t0, whereas they continuously deceased with ALK, PECT1, and CELL feeding at both t24 and t48 compared to t0, while feeding CON and PECT2 decreased the relative abundances of Bacteroidetes at t24, which slightly increased again at t48. The relative abundance of Firmicutes in ALK, CON, PECT1, and PECT2 was quite stable during the fiber fermentation period, while it continuously increased in CELL. The relative abundances of Actinobacteria in ALK, PECT1, and PECT2 increased, whereas those of CON and CELL were stable during the fiber fermentation period. ALK decreased the relative abundance of Proteobacteria, while the others treatments increased this phylum during the fiber fermentation period.

Figure 4B and Supplementary Figure S4B show that the most abundant microbes at order level were: Bacteroidales, Clostridiales, Coriobacteriales, and Selenomonadales. The relative abundances of Bacteroidales in response to ALK, CON, PECT1, PECT2, and CELL were quite similar to each other at t0 (as expected), whereas they continuously deceased with ALK, PECT2, and CELL feeding at both t24 and t48 compared to t0, while feeding CON and PECT2 decreased the relative abundances of Bacteroidetes at t24, which slightly increased again at t48. The relative abundance of Clostridiales in ALK, CON, and CELL increased, and that of PECT1 was stable during the fiber fermentation period, whereas Clostridiales continuously deceased with PECT2 feeding at both t24 and t48 compared to t0. ALK, PECT1, and PECT2 increased the relative abundance of Coriobacteriales, while that of CON decreased and that of CELL was stable. The relative abundance of Selenomonadales in CON increased, while those of ALK, PECT1, PECT2, and CELL were quite stable.

Figure 4C and Supplementary Figure S4C show that the different substrates had an effect on relative abundance at the family level, and the top six most abundant families are summarized below. ALK, PECT2, and CELL feeding decreased the relative abundance of Prevotellaceae, Ruminococcaceae, and Ranunculaceae. Lachnospiraceae was stable, while Atopobiaceae and Acidaminococcus increased upon feeding ALK. Lachnospiraceae decreased, Atopobiaceae increased, and Acidaminococcus was stable upon feeding PECT2. Lachnospiraceae increased, Atopobiaceae was stable, and Acidaminococcus decreased with CELL treatment. CON and PECT1 feeding decreased the relative abundance of Prevotellaceae, Lachnospiraceae, and Ranunculaceae, and stabilized Ruminococcaceae and Acidaminococcus. The numbers of Atopobiaceae decreased with CON, while it increased with PECT1.

**FIGURE 5:**
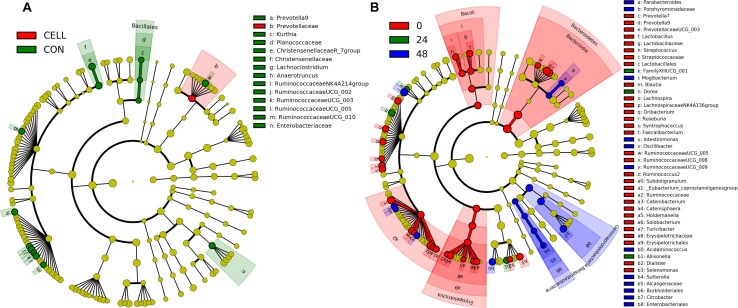
LEfSe results of pig microbiota fed with **(A)** CELL and CON, and **(B)** at time point 0, 24, and 48 h. Differences are represented by the color of the substrates (red, green, and blue indicating CELL and time point 0 h, CON and time point 24 h, and time point 48 h, for panels **(A,B)**, respectively).

Linear discriminant analysis effect size [LEfSe] (Segata et al., 2011) was used to find the significantly different taxonomic ASVs between treatments (Figure 5A and Supplementary Figure S5A) and during the fiber fermentation period (Figure 5B and Supplementary Figure S5B). The relative abundance of Family Prevotellaceae was significantly higher in CELL treatment compared to CON. The relative abundances of ten genera were significant higher in CON compared to CELL: *Prevotella 9*, *Kurthia*, *Christensenellaceae* R_7 group, *Lachnoclostridium*, *Anaerotruncus*, *Ruminococcaceae NK4A214* group, *Ruminococcaceae UCG*_002, and *Ruminococcaceae UCG*_003, *Ruminococcaceae UCG*_005, and *Ruminococcaceae UCG*_*010*. There were no significantly different microbes in PECT1 and PECT2 treatments.

More significantly different microbes were detected among the different time points (Figure 5B and Supplementary Figure S5B). At genus level, the relative abundance of three microbes, *Dorea*, *Allisonella*, and *FamilyXIIIUCG_001* (in the order of Clostridiales), were significantly increased after 24 h adaptation. The relative abundance of eight microbes were significantly higher in t48 compared to the earlier time points. These were *Parabacteroides*, *Mogibacterium*, *Intestinimonas*, *Oscillibacter*, *RuminococcaceaeUCG_009*, *Acidaminococcus*, *Sutterella*, and *Citrobacter*. Figure 5B shows that the relative abundance of 24 genera (linked to t0) significantly decreased after the fiber adaptation period (t24 and t48).

### Functional Metagenomic Profiles Changed After the Fiber Fermentation Period

**FIGURE 6:**
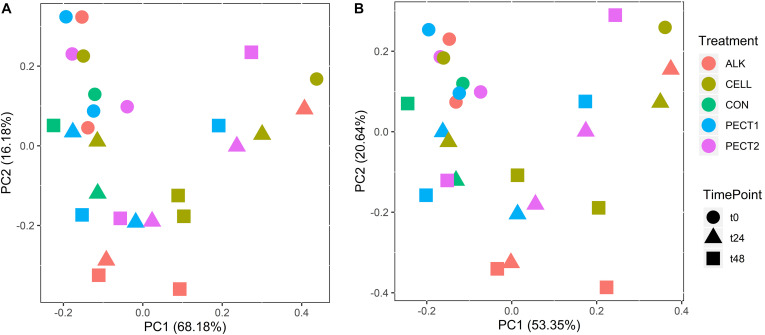
PCA plots of the relative abundances of Enzyme Classification numbers **(A)** and pathways **(B)** from microbiota fed with CELL, CON, PECT1, PECT2, and ALK at different time points (0, 24, and 48 h).

The relative abundances of PICRUSt2-predicted Enzyme Classification numbers and pathways were used to create PCA plots (Figure 6). Figure 6 shows that samples from t0 clustered together and clearly separated with samples from t24 and t48. However, there was no clearly separation (*P* > 0.05) among different treatments (Figure 6).

**FIGURE 7:**
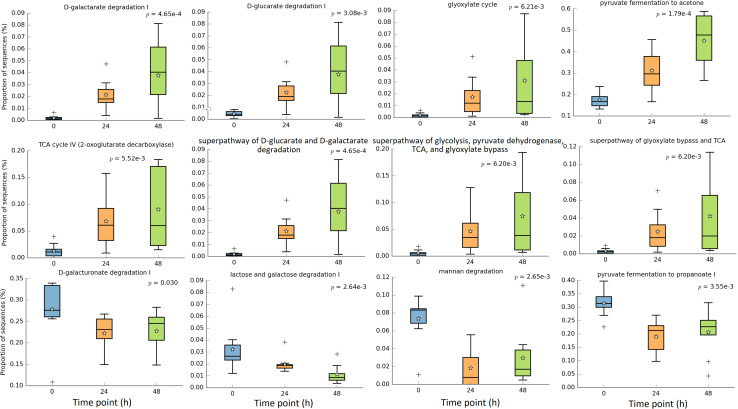
Significantly different metagenomic functions in relative abundance among different time points.

In this study we focused on carbohydrate metabolism related microbial functions. Figure 7 shows that 8 pathways were significantly increased after the fiber adaptation period, and they were D-galactarate degradation I, D-glucarate degradation I, glyoxylate cycle, pyruvate fermentation to acetone, TCA cycle IV (2-oxoglutarate decarboxylase), superpathway of “D-glucarate and D-galactarate degradation”, superpathway of “glycolysis, pyruvate dehydrogenase, TCA, and glyoxylate bypass”, and superpathway of “glyoxylate bypass and TCA”. On the other hand, 4 pathways were significantly decreased after the fiber adaptation period, which were D-galacturonate degradation I, lactose and galactose degradation I, mannan degradation, and pyruvate fermentation to propanoate I. Additional significant increased other pathways and all enzymes are displayed in Supplementary Tables S3, S4, respectively. There were no significantly different pathways between treatments and CON.

### The Cumulative Amount of SCFA Continuously Increased During the Fiber Fermentation Period

**FIGURE 8:**
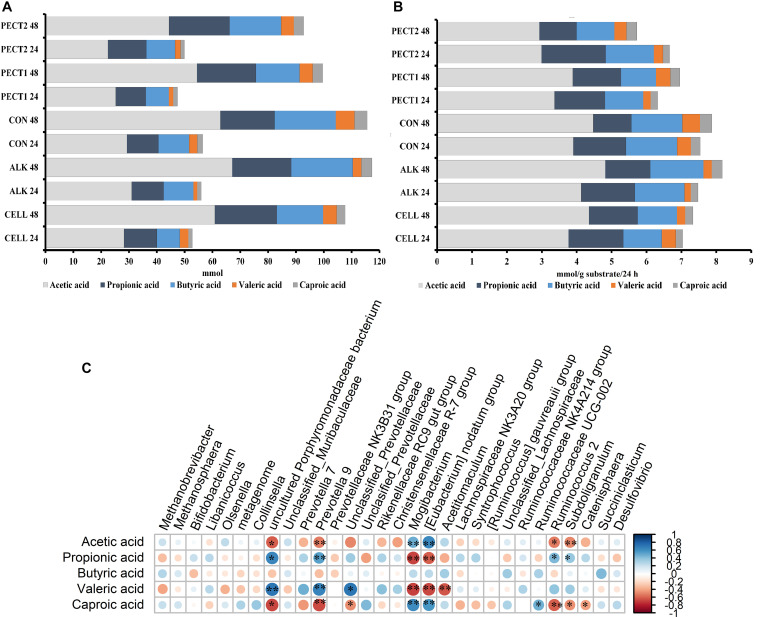
Average cumulative short chain fatty acid (SCFA) production in mmol **(A)** and SCFA increase in mmol/g substrate/24 h **(B)** for non-processed RSM (CON) and RSM processed by Accellerase 1000 (CELL), Pectinex Ultra SP (PECT1), Multifect Pectinase (PECT2), or 6 M NaOH (ALK). The values at time point 0 were artificially set to 0 to allow determination of cumulative production. **(C)** correlation between SCFA production and core genera, *p < 0.05; blue: positive correlation; red: negative correlation. Core genera: the table was normalized via division by the sum of sequences in a given sample and multiplied by the minimum sum across all samples. Relative abundances were filtered as follows: values below a relative abundance threshold of 0.01% were not taken into account; taxa with a median relative abundance < 1% in all groups were not considered for statistical analysis.

Figure 8A shows the average cumulative amount of total, individual SCFA (acetic, propionic, butyric, and including the middle chain fatty acids valeric and caproic acid) at t24 and t48. When mentioning total SCFA or SCFA production, the middle chain fatty acids valeric and caproic acid, were included in the current context. Production of total and individual SCFA constantly increased after 24 and 48 h of fermentation. As generally observed, more acetic acid was produced compared to the amount of propionic and butyric acid, which were similar to each other. There were no significant difference between the first 24 h (t24) and second 24 h (t48) in SCFA production, except for propionic acid production, where the amount of propionic acid production at t24 was significant higher than that at t48 (*P* < 0.05), independent of substrate (Figure 8B). The first 24 h of fiber adaptation resulted in an increase of 7.0, 7.4, 7.5, 6.3, and 6.6 mmol total SCFA/g substrate in response to ALK, CELL, CON, PECT1, and PECT2, respectively. This slightly increased during the second 24 h of fermentation, to 7.1, 8.2, 7.9, and 7.0 mmol total SCFA/g substrate in response to ALK, CELL, CON, and PECT1 feeding, respectively. Only for PECT2 the SCFA production slightly decreased during the second 24 h of fermentation compared to the first 24 h (5.7 mmol SCFA/g substrate).

There were no significant differences in SCFA production between unprocessed and processed RSM during the fiber fermentation period.

Correlations between the relative abundance at genus level and SCFA production were evaluated (Figure 8C). *Prevotella* 9 (*P*_*propionic acid*_ < 0.01, *P*_*valeric acid*_ < 0.01) and uncultured *Porphyromonadaceae bacterium* (*P*_*propionic acid*_ = 0.03, *P*_*valeric acid*_ < 0.01) had significant positive correlations with both propionic and valeric acid, while *Mogibacterium* (*P*_*propionic acid*_ < 0.01, *P*_*valeric acid*_ < 0.01) and [*Eubacterium*] *nodatum* group (*P*_*propionic acid*_ = 0.02, *P*_*valeric acid*_ < 0.01) had negative correlation with these acids. Caproic acid significantly negatively correlated with *Prevotella* 9 (*P* < 0.01), *Ruminococcus* 2 (*P* = 0.03), uncultured *Porphyromonadaceae bacterium* (*P* = 0.01) and *Catenisphaera* (*P* = 0.04), whereas *Mogibacterium* (*P*_*acetic acid*_ = 0.01, *P*_*caproic acid*_ = 0.03) and [*Eubacterium*] *nodatum* group (*P*_*acetic acid*_ = 0.01, *P*_*caproic acid*_ = 0.03) positively correlated with acetic and caproic acid. An unclassified genus from Prevotellaceae (*P* < 0.01) had positive correlation with valeric acid, while *Acetitomaculum* (*P* < 0.01) negatively correlated with it. *Subdoligranulum* significantly negatively correlated with acetic (*P* < 0.01) and caproic acid (*P* = 0.02), while it had positive correlation with propionic acid (*P* = 0.04).

## Discussion

The pig gut microbiota needs an adaptation period to express its maximum enzymatic potential after a change of diet (Castillo et al., 2007). In the current study, treatment of RSM was performed prior to ingestion to decrease this adaptation period, but no significant difference was detected in SCFA production among non-processed (CON) and processed RSM (ALK, PECT1, PECT2, and CELL) fermentation during the 48 h fiber fermentation period in the swine *in vitro* large intestine model (SLIM). However, when another 24 h fermentation period was performed after this 48 h ‘fiber adaptation’ period, considerable changes were found in the amount of SCFA production in response to different treatments (Long et al. manuscript submitted). Previous studies show that treatments (ALK, PECT1 and PECT2) on RSM did improve the NSP degradation after 4-week adaptation in boilers and pigs (De Vries et al., 2014; Pustjens et al., 2014a). Thus, it is important to know how the microbiota changes during the fiber adaptation period. The current study showed that essentially a 48 h period was sufficient to increase the pathways, involved in carbohydrate fermentation.

Alpha-diversity significantly decreased after 48 h RSM fermentation, while there were no significant changes between treatments and CON during the fiber adaptation period. This may due to the selection of particular microbes to utilize RSM regardless of the treatment, since RSM was the major nutrient in all cases, compared to the microbial adaptation period (16 h SIEMP adaptation), and the different treatments applied only led to changes in cell wall structures in RSM (Pustjens et al., 2014b). In previous studies, it has been shown that RSM increased the relative abundance of *Dorea* and *Lactobacillus* in chickens (Chiang et al., 2009; Long et al., 2017). *Lachnospira*, *Coprococcus*, *Bulleidia*, and *Shuttleworthia* are increased by RSM after three weeks adaptation in pigs (Umu et al., 2018). In the current study, a large number of genera (24 genera) significantly decreased after the fiber fermentation period, and only three and eight genera were considerably increased after adapting to the substrate for 24 and 48 h, respectively. The decreased taxa may not utilize RSM well, while RSM may be beneficial for the increased taxa. The significantly increased genera observed in the current study are *Dorea*, *Allisonella*, *FamilyXIIIUCG_*001 (in the order of Clostridiales), *Parabacteroides*, *Mogibacterium*, *Intestinimonas*, *Oscillibacter*, *Sutterella, Citrobacter*, *RuminococcaceaeUCG_*009, and *Acidaminococcus*. No overlapping genera were observed between the current study and research of Umu et al. (2018), which might be because of a different pig breed. On the other hand, *Dorea* was found significantly increased by RSM in both the current study and research of Long et al., where six weeks of adaptation period was performed in laying hens (Long et al., 2017). This indicated that *Dorea* might be a potential RSM degrader.

Disappearance of some low abundance microbes were observed in either processed or non-processed RSM treatment since unweighted UniFrac (only considering presence or absence) was found significantly different (*P* = 0.02), while there was no difference in terms of weighted UniFrac (accounting also for abundance of observed organisms) among the treatments. This indicated that the treatments applied on RSM indeed have an effect on the microbiota composition, but that the short fiber fermentation period used may not yet have led to significant changes. In another study, we showed, however, that weighted UniFrac was observed to be significantly different among the treatments when a single shot of the fibers was introduced after this initial 48 h (Long et al. manuscript submitted). Zooming in to individual taxa, at the family level, Prevotellaceae significantly increased with CELL. Prevotellaceae is an important family, which is involved in degrading hemicellulose (Emerson and Weimer, 2017). We also observed that genera (e.g., *Prevotella* 9) from Prevotellaceae significantly positively correlated with SCFA production (Figure 7). To demonstrate the dynamic changes in swine microbiota composition in response to differently pretreated RSM, lumen samples from each time point were analyzed. The most dominant phyla were Firmicutes, Bacteroidetes, and Actinobacteria at t0, regardless of treatment (Figure 4). This was in line with previous studies (Casellas et al., 2014; Costa et al., 2014; Ramayo-Caldas et al., 2016; Cantu-Jungles et al., 2019), which indicated that core microbes existed. The relative abundance of Bacteroidetes decreased after supplementing with ALK, PECT1, and PECT2, whereas that of Actinobacteria increased (Figure 4). These observations implied that supplementation of ALK, PECT1, and PECT2 stimulated the growth of microbes from phylum Actinobacteria and inhibited the growth of bacteria from phylum Bacteroidetes. Actinobacteria has been reported to efficiently utilize (hemi)cellulose (Munk et al., 2009; Rastogi et al., 2010; Wang et al., 2016). Firmicutes and Proteobacteria were stimulated after supplementing with CELL (Figure 4). Firmicutes is a phylum that consists of a group of dominant butyrate-producing bacteria (Sheridan et al., 2016; La Rosa et al., 2019), which play an important role in carbohydrate degradation. Research showed that the less abundant phylum Proteobacteria enriched gene cohorts of glycolytic enzymes in human gut microbiota (Bradley and Pollard, 2017), which indicated that Proteobacteria might play a similar role in utilizing RSM in the current study. Zooming in to family level, ALK, PECT1, and PECT2 simulated the growth of Atopobiaceae (phylum Actinobacteria); CELL benefited the growth of Lachnospiraceae (phylum Firmicutes). The observations above suggested these microorganisms that were stimulated by these treatments play roles in the fiber degradation. Altogether, this indicated that fiber adaptation period competitively excluded low abundant microbes and created a better ecological niche for the growth of RSM-utilization (fiber fermentation) members of the microbiota.

CELL and ALK have larger effect on pig gut microbiota compared to other treatments, since their within-treatment distances between t0 and t48 were larger than those of CON, PECT1, and PECT2 (Figure 3). Our other study, with the fiber shot after 48 h adaptation, showed also that more genera were observed to be significantly changed particularly upon feeding CELL and ALK than with PECT1 and PECT2, when compared to CON (Long et al. manuscript submitted). The primary cell wall of RSM contains a backbone of cellulose microfibrils, which are interlinked with xyloglucan via hydrogen bonds forming a stiff network (Carpita and Gibeaut, 1993). Pectins are linked to each other and cross-linked with hemicellulose and cellulose (Broxterman and Schols, 2018). CELL might have relative easy access to target cellulose in RSM, whereas PECT1 and PECT2 could have less opportunity to access pectins, supposedly hindered by cellulose microfibrils. Cell wall structures processed by CELL would lead to utilization of the fibers by microbes, and increase their abundances. Alkaline pretreatment of RSM breaks the alkali-labile bonds, which are known to hinder the complete fermentation of NSP in swine, and improved NSP utilization in feed (Pustjens et al., 2014b). However, also PECT1 and PECT2 affected the swine gut microbiota in these experiments, since gPCA showed that their distances between time point 0 and 48 h were larger than that of CON. Both gPCA and PCoA are used to classify the dissimilarity between treatments. PCoA might be more often used to classify the dissimilarity between two or more groups, while gPCA can more precisely detect the dissimilarity between two samples based on their distance in the plot. A previous report showed that a cocktail of PECT1 and PECT2 improved degradability of non-starch polysaccharides of RSM in broilers (De Vries et al., 2014). Treatments on RSM, either with carbohydrases or alkaline, did not improve SCFA production during the fiber fermentation period compared to non-processed RSM. This indicated that the processing applied to RSM could not improve their degradability compared to CON, in other words, they might not increase the feed efficiency during this period *in vivo*. It might be the fiber adaptation period was not enough to express its maximum enzymatic potential (Castillo et al., 2007), although as mentioned earlier within 48 h the pathways involved in carbohydrate fermentation as increased (Figure 7). Moreover, our own experiments with the fiber shot after 48 h showed that more pathways related to fiber fermentation were predicted to be upregulated (Long et al. manuscript submitted). PCA plots of Enzyme Classification numbers and pathways also show that samples from t0 clustered together, but separately from samples from t24 and t48. This also indicated that microbial function between t0 and later time points was modulated, and geared toward degradation of these new substrates. Previous studies showed that a high-fiber rapeseed diet did not results in a significant increase in SCFA content in the chyme of RSM-fed pigs after a 3-week adaptation period (Chen et al., 2018; Umu et al., 2018), which seemed to indicate that the adaptation period was not long enough. Thus, strategies should be considered to shorten the fiber adaptation period, in order to increase feed efficiency.

In conclusion, the current study demonstrated that microbiota composition was significantly affected by both RSM (processed or not) and adaptation time. Unweighted UniFrac showed that microbial community composition was significantly separated between processed RSM and CON, and CELL and ALK in general changed the microbiota composition more than PECT1 and PECT2 did. Carbohydrate metabolism related microbial functions were significantly increased after the fiber fermentation period. However, degradability of the processed RSM was not improved compared to CON during the fiber fermentation period, as assessed by SCFA production, which indicated a relative long adaptation period is needed after a diet change to RSM for swine microbiota. Thus, the significantly different microbes detected between treatments, and the bacteria considerably correlating with SCFA production might be an important finding to design specific strategies to shorten the fiber adaptation period, in order to increase feed efficiency in animals, and particularly in pig production.

## Data Availability Statement

The original contributions presented in the study are publicly available. This data can be found here: https://www.ebi.ac.uk/ena/data/view/PRJEB36982.

## Author Contributions

CL, SV, and KV designed the original study, interpreted the data, and revised the work critically. CL acquired and analyzed the data.

## Conflict of Interest

The authors declare that the research was conducted in the absence of any commercial or financial relationships that could be construed as a potential conflict of interest.
