# Supplementary material for: Differently Pre-treated Rapeseed Meals Affect in vitro Swine Gut Microbiota Composition
Source: Front Microbiol. 2020 Aug 28;11:570985. doi: 10.3389/fmicb.2020.570985 (PMC7483658; doi:10.3389/fmicb.2020.570985)
Supplement: Supplementary file 2 [file Data_Sheet_1.docx]

Figure S1 SLIM system. A. peristaltic compartments; B. pH-electrode; C. alkali pump; D. dialysis liquid circuit with hollow fibers; E. level-sensor; F. N_2_ gas inlet; G. sampling port; H. gas outlet; I. 'ileum efflux' container, or, food syringe; J. temperature sensor.


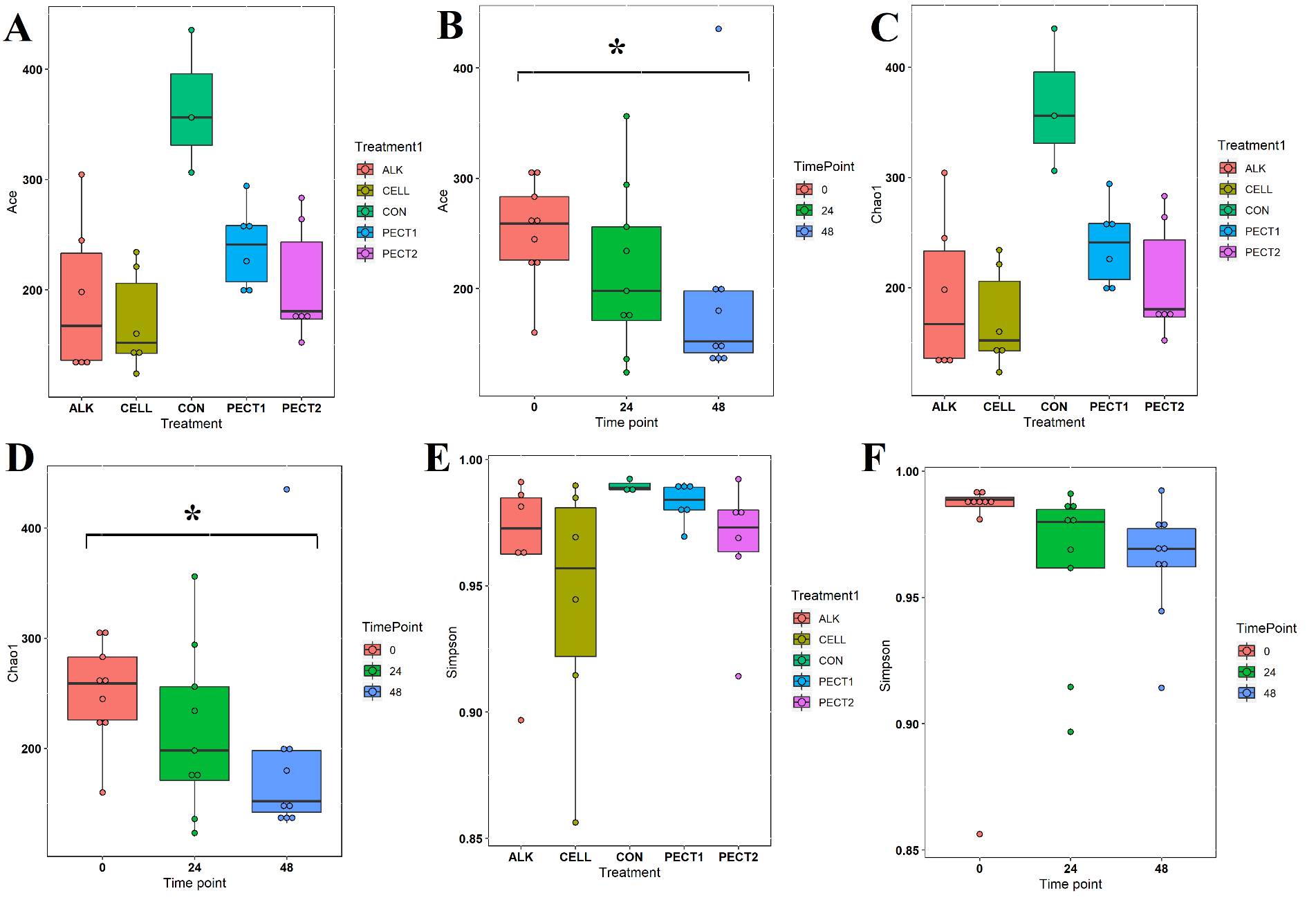


Figure S2. Community α-diversity represented by Ace, Chao2 and Simpson index from each treatment (A, C, F) and time point (B, D, E). The treatments are non-processed RSM (CON) and RSM processed by Accellerase 1000 (CELL), Pectinex Ultra SP (PECT1), Multifect Pectinase (PECT2), or 6M NaOH (ALK).


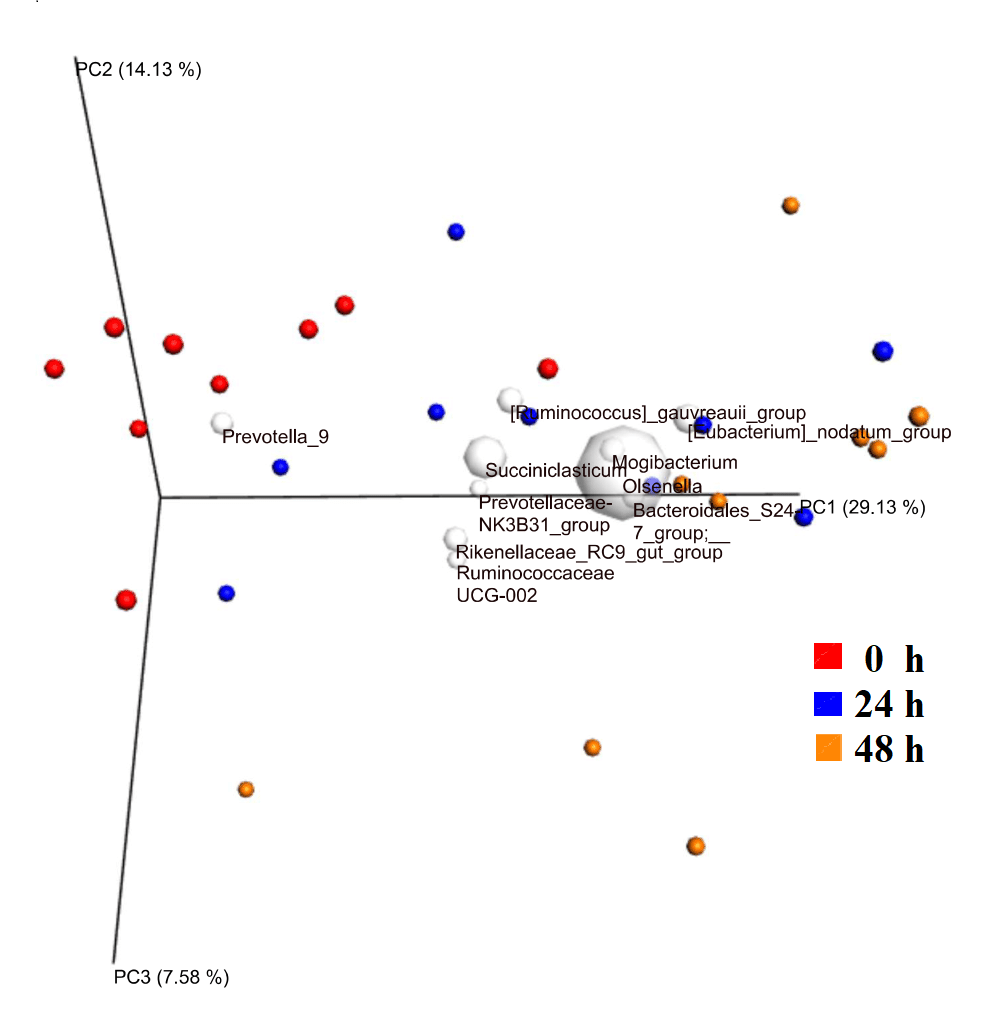


Figure S3. Biplot of unweighted UniFrac. Samples were grouped by color in terms of time point they belonged to: time point 0 h (red), 24 h (blue), 48 h (orange).

Figure S4. Relative abundances of microbial phyla (A), orders (B) and families (C) in pig microbiomes fed with non-processed RSM (CON) or RSM processed by Accellerase 1000 (CELL), Pectinex Ultra SP (PECT1), Multifect Pectinase (PECT2), or 6M NaOH (ALK) at different time points (0 h, 24 h, and 48 h).


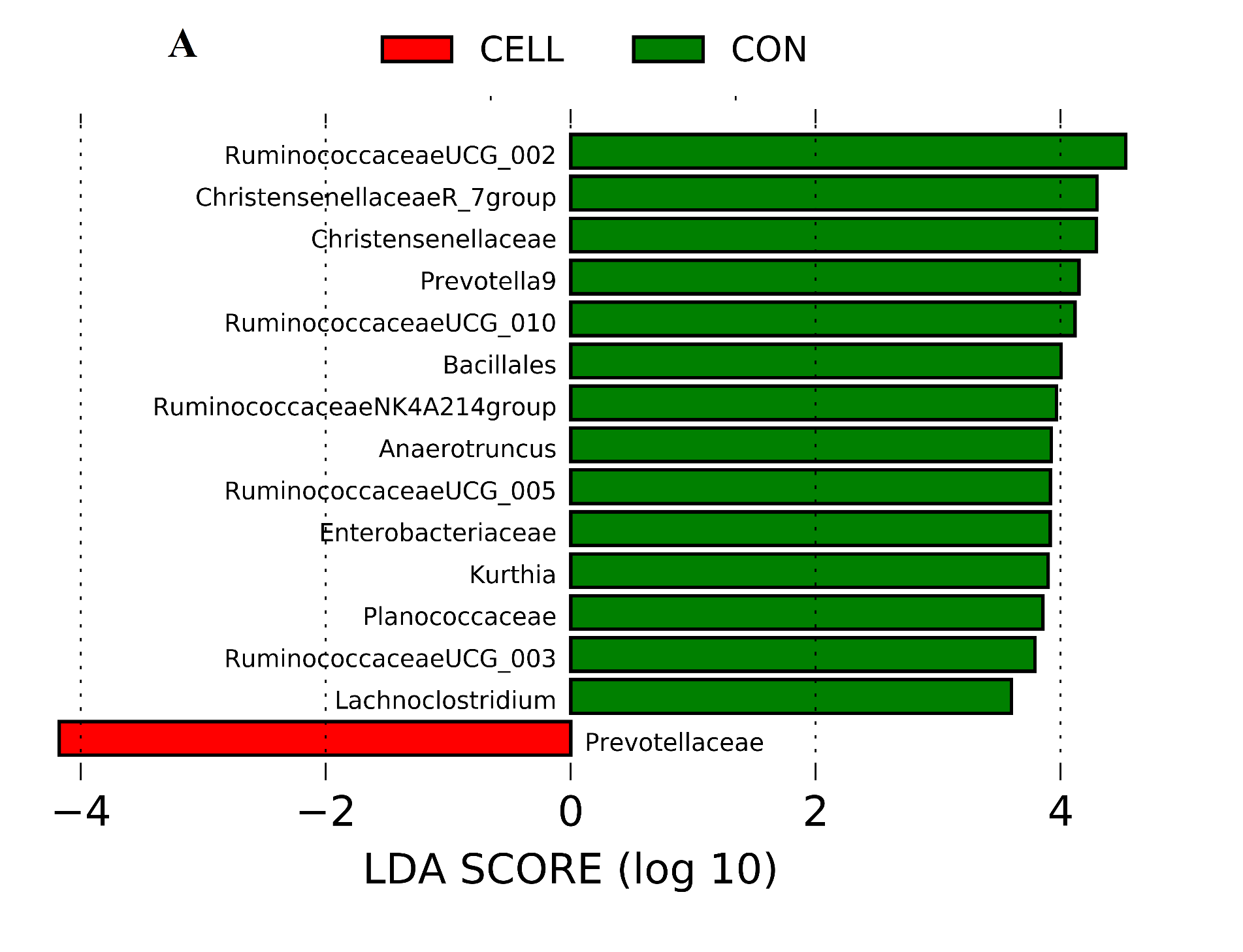

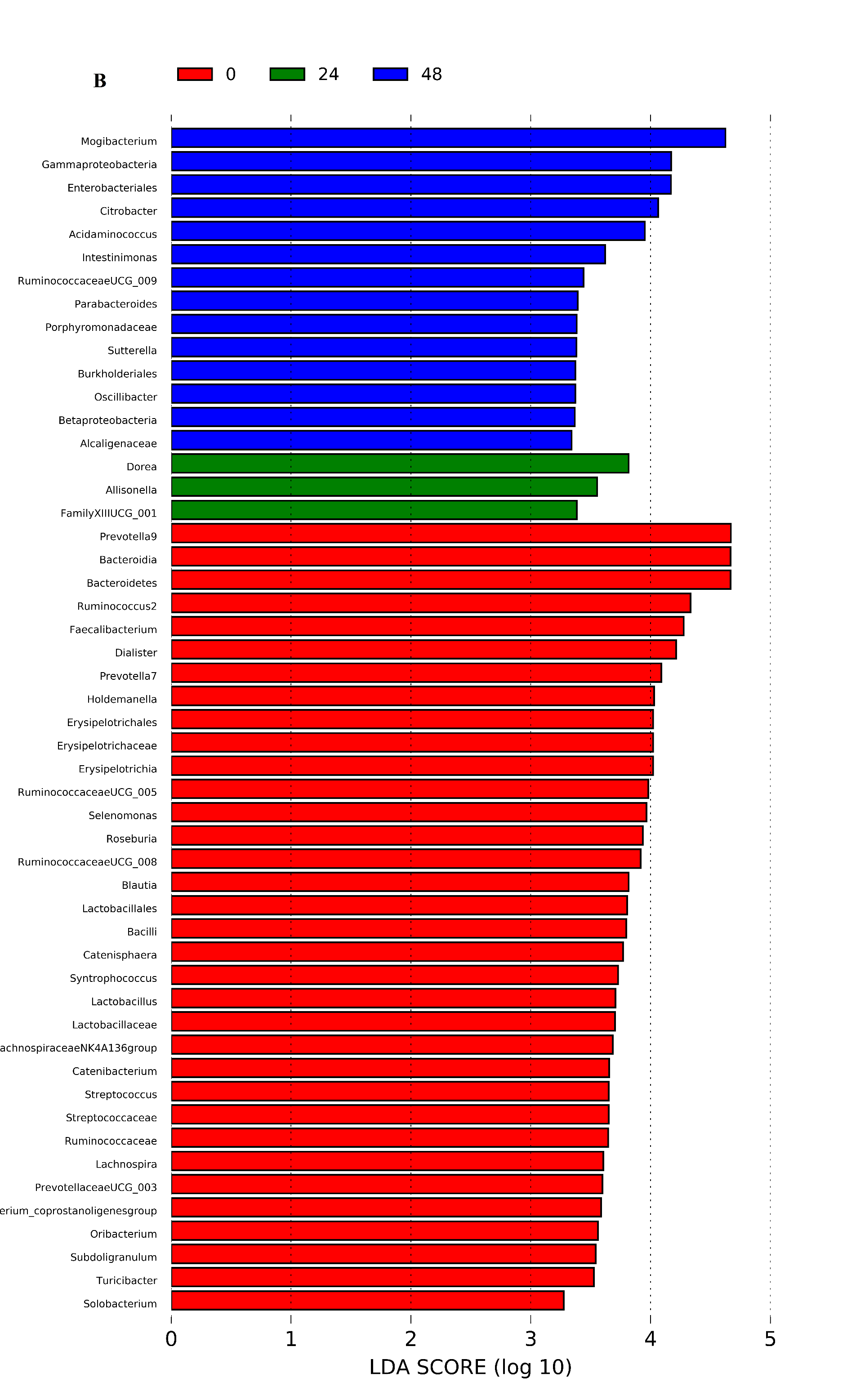


Figure S5. LEfSe results of pig microbiota fed with (A) CELL and CON, and (B) at time point 0, 24, and 48 h.
